# Discovery of Rhubarb Anthraquinones Physcion and Rhein as Functional Inhibitors of TRPV1 Against Lipopolysaccharide-Induced Neuroinflammation

**DOI:** 10.3390/molecules31122049

**Published:** 2026-06-11

**Authors:** Shuli Li, Yulin He, Hiotong Kam, Hanbin Chen, Jin-Song Bian, Nan Xu, Guiyi Gong, Qiwen Liao, Chen Zhao, Binrui Yang, Liang Chen, Kit Ieng Kuok, Simon Ming Yuen Lee

**Affiliations:** 1 State Key Laboratory of Mechanism and Quality of Chinese Medicine, Institute of Chinese Medical Sciences, University of Macau, Taipa, Macao 999078, China; yc27521@um.edu.mo (S.L.); yc17526@um.edu.mo (N.X.); 2 Department of Food Science and Nutrition, The Hong Kong Polytechnic University, Hung Hom, Kowloon, Hong Kong 999077, China; yulinhe@suse.edu.cn (Y.H.); yb97508@um.edu.mo (H.K.); guiyi.gong@polyu.edu.hk (G.G.); qiwen.liao@polyu.edu.hk (Q.L.); yc17509@um.edu.mo (C.Z.); 3 School of Bioengineering, Sichuan University of Science and Engineering, Yibin 644000, China; 4 Joint Laboratory of Guangdong-Hong Kong Universities for Vascular Homeostasis and Diseases, Department of Pharmacology, School of Medicine, Southern University of Science and Technology, Shenzhen 518055, China; chenhb@sustech.edu.cn (H.C.); bianjs@sustech.edu.cn (J.-S.B.); 5 PolyU-BGI Joint Research Centre for Genomics and Synthetic Biology in Global Ocean Resources, The Hong Kong Polytechnic University, Hung Hom, Hong Kong 999077, China; 6 Nutrilite Health Institute, Amway (Shanghai) Innovation & Science Co., Ltd., Shanghai 201203, China; binrui.yang@amway.com (B.Y.); clark.chen@amway.com (L.C.); 7 Crystal Vision Biotechnology Limited, Taipa, Macao 999078, China; kuokkitieng@gmail.com

**Keywords:** rhubarb, anthraquinones, physcion, rhein, neuroinflammation, TRPV1

## Abstract

Neuroinflammation, mediated by microglia and astrocytes, is an abnormal immune reaction in central nervous system (CNS) disorders. Stimulation of TRPV1 has been found to enhance microglial activation, resulting in a pro-inflammatory response. Natural anthraquinones such as physcion and rhein are commonly found in rhubarb, a medicinal plant recognized for its dual role in culinary and therapeutic applications. The therapeutic potential and mechanisms of these anthraquinones remain largely unexplored. This research aims to examine how anthraquinones protect against neuroinflammation and delineate the underlying mechanisms in lipopolysaccharide (LPS)-mediated cellular and zebrafish models. Among the representative anthraquinone analogs, physcion and rhein showed potent functional inhibitory activity against the TRPV1 channel. The production of nitric oxide (NO) and secretion of pro-inflammatory factors triggered by LPS were significantly reduced in BV-2 cells through regulation of *iNOS*, *IL-6*, *IL-1β*, and *TNF-α* mRNA expression. Moreover, physcion and rhein inhibited calcium influx and exerted anti-neuroinflammatory effects, which were closely associated with the suppression of Ca^2+^/CAMKK2/AKT and the PI3K/AKT-mediated NF-κB activation pathways. Furthermore, physcion and rhein reduced LPS-driven neutrophil recruitment to the brain and ameliorated locomotor deficits in zebrafish larvae, with the restoration of *IL-1β*, *IL-6*, and *TNF-α* transcript levels to baseline. In conclusion, natural-derived anthraquinones from rhubarb, physcion and rhein, acted as functional inhibitors of TRPV1-mediated calcium dynamics and significantly reduced LPS-mediated neuroinflammation in microglial cells and zebrafish larvae, suggesting promise as therapeutics for neurological disorders.

## 1. Introduction

Neuroinflammation represents a generalized immune reaction in the CNS, triggered when peripheral inflammation engages the blood–brain barrier, glial cells, and neurons [1]. This mechanism plays a pivotal role in neurodegeneration and is associated with the course of several nervous system disorders [2,3]. To effectively address overactivated neuroinflammatory signaling in the treatment of neurodegenerative conditions, a thorough investigation into the cellular and molecular mechanisms driving neuroinflammation is critical [4]. As the dominant innate immune cells, microglia are dispersed across the brain and serve as the initial responders to CNS injuries [5,6]. As microglia remain activated for a long time, the release of cellular factors and neurotrophic molecules could lead to prolonged neurodegenerative changes leading to triggered neurodegenerative diseases [7].

TRPV1, a member of the vanilloid receptor subfamily of TRP proteins, has been shown to be broadly expressed throughout the CNS [8], exerting a pathogenic role in lots of neurological disorders [9,10,11]. The TRPV1 channel is well validated for being expressed in glial cells and it is highly penetrant for calcium ions in microglia, promoting calcium ion (Ca^2+^) influx, thereby affecting cell reactions regarding migration, generation of reactive oxygen species (ROS) and inflammatory signaling molecules [12,13]. The TRPV1 channel contributes to neuroinflammatory regulation, with its activation amplifying microglia-mediated inflammation through the engagement of specific signaling cascades consequently leading to pro-inflammatory responses [14]. It was reported that capsazepine (CPZ), a TRPV1 antagonist, decreased TNF-α and IFN-γ release from the innate response to immunity and suppressed neurotoxicity [15]. Ca^2+^ orchestrates numerous cellular responses, including those involved in neuroinflammation [16]. Elevated intracellular Ca^2+^ stimulates CAMKK2, and functions as a pivotal mediator for transmitting downstream signaling cascades [17,18]. While CAMKK2 can directly activate AKT independently of PI3K [19], the PI3K/AKT axis itself is responsive to calcium signaling [20,21].

Naturally occurring anthraquinones including rhein, physcion, emodin, aloe-emodin and chrysophanol are commonly found active ingredients in food and medicinal plants such as rhubarb [22]. Rhubarb has gained worldwide recognition for its roles in both culinary and medicinal contexts [23]. While its edible petioles are prized for their tart flavor in desserts, jams, and beverages [24], various pharmacological effects of these anthraquinones have been reported, including inflammation inhibition [25], tumor suppression [26,27] and neuroprotection [28].

Currently, conventional therapies for neuroinflammatory conditions often suffer from limited long-term efficacy and significant systemic side effects. Furthermore, while TRPV1 has emerged as a promising therapeutic target for neuroprotection [29], the clinical application of synthetic TRPV1 antagonists remains challenging because they frequently induce severe adverse events, particularly hyperthermia and impaired thermal sensation [30]. Therefore, there is an urgent unmet clinical need to discover novel TRPV1 modulators with improved safety profiles. Naturally derived anthraquinone compounds, such as physcion and rhein, present a highly attractive alternative. Compared to existing synthetic treatments, their unique structural scaffolds may offer a wider therapeutic window, potentially achieving a more favorable pharmacological balance between effective functional TRPV1 modulation and cellular safety.

This work focused on elucidating their molecular targets and underlying mechanisms on the anti-neuroinflammatory effects of selected natural anthraquinones in HEK293-hTRPV1 cells and the LPS-elicited neuroinflammatory process in BV-2 microglia cells and Tg(*mpo*:EGFP) zebrafish.

## 2. Results

### 2.1. Molecular Docking of Anthraquinones Against TRPV1 Channel

A preliminary virtual screening was conducted on a small-molecule library, with the top-tier candidates prioritized for further investigation based on their binding affinities (Supplementary Table S1). Cluster analysis of these compounds revealed that several belonged to the rhubarb anthraquinones. A focused panel of five rhubarb-derived derivatives, including physcion and rhein alongside emodin, chrysophanol, and aloe-emodin, was investigated to elucidate their TRPV1-modulatory potential. Their binding thermodynamics were subsequently rigorously assessed through the Uni-GBSA algorithm, ensuring a high-fidelity ranking of their inhibitory affinities.

The binding free energies of the five selected anthraquinone compounds were calculated using Uni-GBSA, and their binding affinities to TRPV1 were ranked. The binding free energy of rhein, physcion, chrysophanol, aloe-emodin and emodin was −47.32 kcal/mol, −46.37 kcal/mol, −44.50 kcal/mol, −41.10 kcal/mol and −39.40 kcal/mol respectively. Generally, it is considered that low binding energies reflect stable complexes, with familiar binding sites suggesting functional similarity to the active compounds [31]. The results of the calculations, presented in Table 1, showed that rhein and physcion exhibited lower binding free energies, indicating stronger interactions with TRPV1. The detailed binding free energy values and rankings are summarized in Table 1.

### 2.2. Physcion and Rhein Inhibited Calcium Influx on HEK293-hTRPV1 Cells

**Figure 1:**
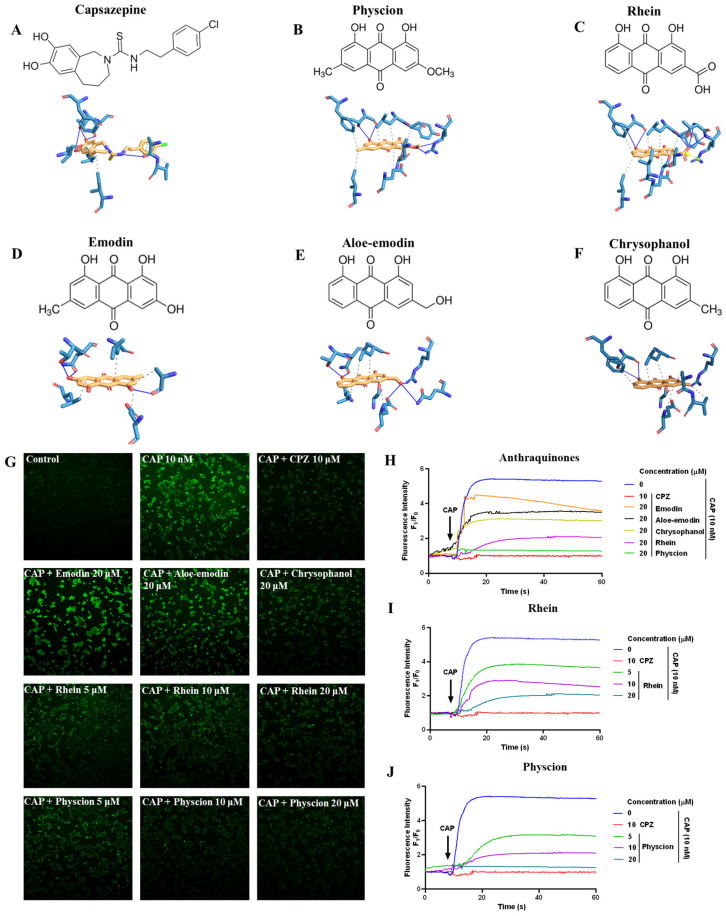
Molecular docking of compounds with TRPV1 and the inhibitory effects of anthraquinones on calcium influx on the HEK293-hTRPV1 cells. (**A**–**F**) The chemical structures of the compounds and a molecular docking visualization of the compounds against TRPV1. The blue structures represent the compounds, the central yellow structure represents TRPV1, hydrophobic interactions and hydrogen bonds are denoted by black dashed and blue solid lines, respectively. (**G**–**J**) Intracellular calcium dynamics in the HEK293-hTRPV1 cells (*n* = 5). (**G**) Typical fluorescence micrographs illustrating cytosolic calcium levels under stimulation with CAP or CPZ. (**H**) Real-time fluorescence intensity traces for five anthraquinones at 20 µM. (**I**,**J**) Concentration-dependent (5, 10 and 20 µM) calcium transients induced by physcion (**I**) and rhein (**J**).

The consensus binding postures of the molecules were obtained by Protein-Ligand Interaction Profiler and visualized by Pymol v1.8. The binding sites of all compounds on TRPV1 were shown in Figure 1A–F. To validate computational predictions, we monitored intracellular calcium mobilization in HEK293-hTRPV1 cells. Prior to these functional evaluations, CCK-8 assays confirmed that all five anthraquinones exhibited minimal cytotoxicity in this cell line, thereby establishing a safe working concentration window (Supplementary Figure S1). This functional assay confirmed the ligands’ modulatory effects on TRPV1-mediated cationic flux. Capsaicin (CAP) is a classical TRPV1 agonist that activates the channel and increases intracellular calcium concentration. At 10 nM, CAP induced a significant calcium influx in cells. Pretreatment with the standard TRPV1 antagonist CPZ (10 µM), utilized as a positive control, significantly suppressed the CAP-evoked calcium influx. While emodin, aloe-emodin, and chrysophanol exhibited only weak inhibitory effects on the fluorescence intensity, physcion and rhein significantly suppressed the CAP-evoked calcium influx in a concentration-dependent manner at 5, 10, and 20 µM (Figure 1G–J). Quantitative analysis revealed that at 20 µM, physcion suppressed the CAP-induced calcium influx by approximately 92%, indicating a near-complete functional suppression. Meanwhile, rhein reduced the peak response by approximately 85%, demonstrating a substantial partial inhibition. Consequently, physcion and rhein were identified as potent functional TRPV1 inhibitors and established as the primary candidates for subsequent in vitro and in vivo neuroinflammation studies.

### 2.3. Physcion and Rhein Suppressed LPS-Induced NO Production in BV-2 Microglia Cells

**Figure 2:**
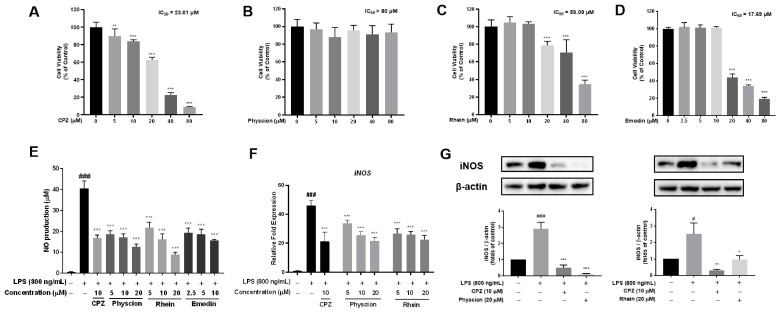
The inhibitory effects of anthraquinones on NO production in LPS-stimulated BV-2 microglia. (**A**–**D**) The evaluation of cell viability following a 24 h incubation with CPZ, physcion, rhein, and emodin via CCK-8 assay (*n* = 5). (**E**) The quantification of NO secretion utilizing a specialized detection kit (*n* = 3). (**F**,**G**) The assessment of iNOS induction at the transcriptional and translational levels. The quantitative data are presented as a percentage of the control (*n* = 3). The quantitative results are expressed as mean ± SD, and the statistical significance of the results was analyzed by one-way ANOVA. ### *p* < 0.001 and # *p* < 0.05 versus the control group; *** *p* < 0.001, ** *p* < 0.01 and * *p* < 0.05 versus the LPS-treated group.

To evaluate the anti-neuroinflammatory activities of physcion, rhein and emodin anthraquinones, we first assessed compound cytotoxicity by CCK-8 assay, and subsequently measured NO secretion while assessing iNOS abundance at both the gene and protein levels. Treatment with physcion did not result in notable lethality within the concentration range from 5 to 80 µM (Figure 2B). The IC_50_ (half-maximal inhibitory concentration) of physcion was higher than 80 µM. The IC_50_ of CPZ, rhein and emodin were 23.0 µM, 59.0 µM and 17.7 µM, respectively (Figure 2A,C,D). Emodin was included as a reference compound in the cellular assays because it is a widely studied rhubarb anthraquinone with known anti-inflammatory properties. While emodin was identified as the most cytotoxic agent in BV-2 cells, the NO quantification assay confirmed that CPZ along with physcion, rhein, and emodin each exerted distinct inhibitory effects on LPS-induced inflammatory signaling, as evidenced by reduced nitrite levels (Figure 2E). Based on these results, compared with emodin, physcion and rhein showed less toxicity towards microglia BV2 cells and exhibited significant inhibition of NO production. Thus, physcion and rhein were advanced to further mechanistic studies based on their overall superiority in target binding affinities, suppression of TRPV1-mediated calcium influx and therapeutic safety windows. Data from qRT-PCR and immunoblotting revealed that both compounds effectively abrogated LPS-triggered iNOS induction, significantly lowering both transcriptional abundance and protein accumulation (Figure 2F,G).

### 2.4. Physcion and Rhein Suppressed Pro-Inflammatory Cytokine Release and mRNA Expression in LPS-Activated BV-2 Microglia Cells

**Figure 3:**
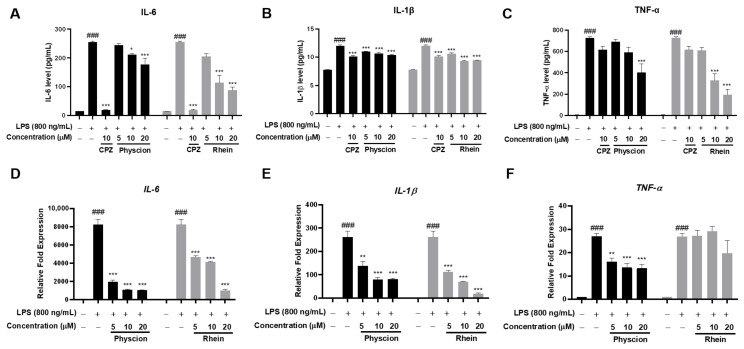
The effect of physcion and rhein on LPS-activated pro-inflammatory cytokine release and gene expression in BV-2 microglia cells. (**A**–**C**) The quantification of secreted pro-inflammatory mediators via ELISA (*n* = 3). (**D**–**F**) Transcriptional analysis of cytokine markers normalized to β-actin as the internal control (*n* = 3). The quantitative results are expressed as mean ± SD; the statistical significance of the results was analyzed by one-way ANOVA. ### *p* < 0.001 versus the control group; *** *p* < 0.001, ** *p* < 0.01 and * *p* < 0.05 versus the LPS-treated group.

BV-2 microglia cells stimulated with LPS release significant extracellular pro-inflammatory cytokines; thus the suppression of cytokine synthesis and secretion represents a pivotal benchmark for defining therapeutic potential against neuroinflammation. While LPS stimulation induced substantial cytokine release, treatment with graded doses of physcion, rhein, and CPZ markedly suppressed the accumulation of IL-6, TNF-α and IL-1β (Figure 3A–C). These findings were corroborated at the gene expression level, where physcion and rhein demonstrated inhibitory potencies similar to CPZ in abrogating mRNA upregulation (Figure 3D–F). The results indicated that physcion and rhein could downregulate pro-inflammatory cytokine release and gene expression, thereby suppressing the inflammatory response.

### 2.5. Physcion and Rhein Downregulated Calcium-Dependent Pathway in LPS-Activated BV-2 Microglia Cells

**Figure 4:**
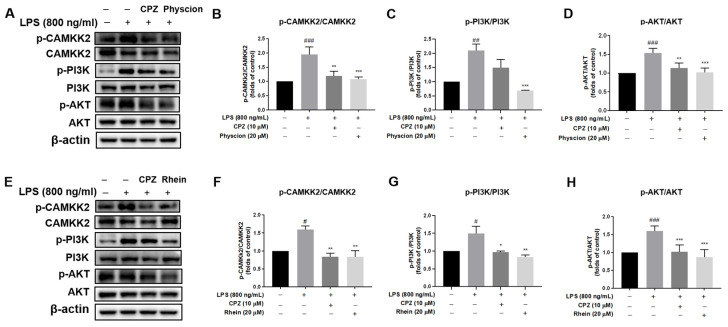
The modulation of the Ca^2+^-dependent CAMKK2/Akt axis by physcion and rhein in LPS-challenged BV-2 microglia. (**A**,**E**) The immunoblotting analysis of signal transduction proteins in the activated microglial model. The expression of p-CAMKK2/CAMKK2 (**B**,**F**), p-PI3K/PI3K (**C**,**G**), p-AKT/AKT (**D**,**H**) were quantified by densitometry (*n* = 3). The quantitative results are expressed as mean ± SD; the statistical significance of the results was analyzed by one-way ANOVA. ### *p* < 0.001, ## *p* < 0.01 and # *p* < 0.05 versus the control group; *** *p* < 0.001, ** *p* < 0.01 and * *p* < 0.05 versus the LPS-treated group.

We investigated the molecular mechanisms of physcion and rhein by determining the protein levels of key TRPV1-regulatory signaling molecules (CAMKK2, PI3K, AKT) using Western blot analysis. Compared to the control, the LPS-treated group exhibited an upregulation in the activation states of CAMKK2, PI3K, and AKT (Figure 4B–D). Notably, physcion and rhein markedly attenuated the phosphorylation of these signaling molecules, suggesting that the therapeutic efficacy of these compounds is mediated via the suppression of the CAMKK2-dependent AKT pathway.

### 2.6. Physcion and Rhein Attenuated LPS-Stimulated Activation of the NF-κB Pathway in BV-2 Microglial Cells

**Figure 5:**
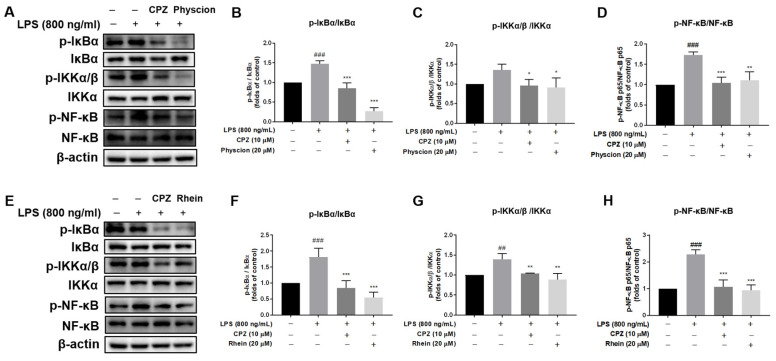
Physcion and rhein attenuated the LPS-stimulated activation of the NF-κB pathway in the BV-2 microglial cells. (**A**,**E**) Representative immunoblots illustrating the protein expressions of key inflammatory mediators following LPS provocation and compound intervention. The expression of p-IκBα/IκBα (**B**,**F**), p-IKKα/β/IKKα (**C**,**G**), p-NF-κB/NF-κB (**D**,**H**) were quantified by densitometry (*n* = 3). The quantitative results are expressed as mean ± SD; the statistical significance of the results was analyzed by one-way ANOVA. ### *p* < 0.001 and ## *p* < 0.01 versus the control group; *** *p* < 0.001, ** *p* < 0.01 and * *p* < 0.05 versus the LPS-treated group.

The pro-inflammatory impact of NF-κB, which operates downstream of the PI3K/Akt pathway, was assessed following intervention with physcion or rhein. In the LPS-activated BV-2 models, Western blot assays confirmed that LPS stimulation promoted a robust phosphorylated state of IκBα, thereby driving the inflammatory response (Figure 5B), IKKα/β (Figure 5C), and NF-κB (Figure 5D), and these hyperphosphorylations were substantially reduced by CPZ, physcion and rhein. These results suggested that the LPS-triggered activation of the IκBα/IKKα/NF-κB cascade was significantly attenuated by treatment with physcion and rhein in the BV-2 cells.

### 2.7. Physcion and Rhein Inhibited LPS-Triggered Neutrophil Recruitment and Attenuated the Induction of Pro-Inflammatory Mediators in Zebrafish Larvae

**Figure 6:**
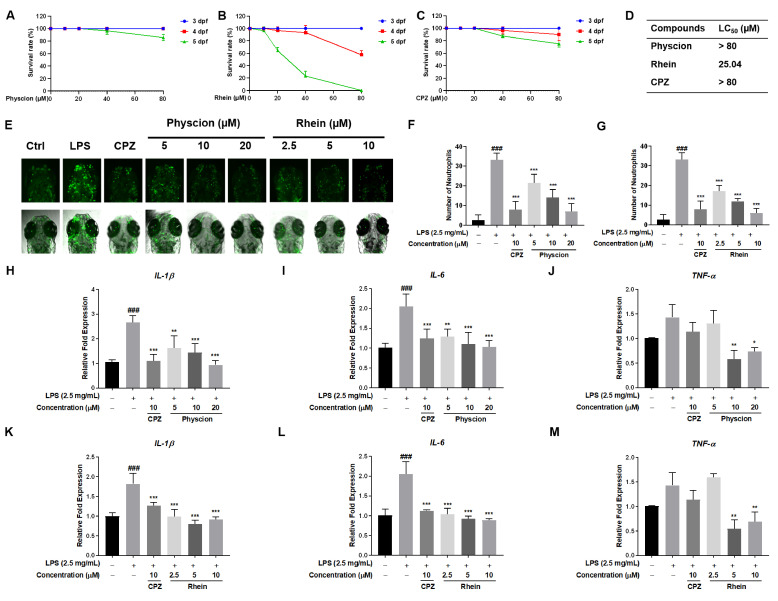
The modulatory effects of physcion and rhein on LPS-triggered neutrophil mobilization and cytokine induction in zebrafish. (**A**–**C**) AB wild-type zebrafish larvae at 3 dpf were allocated into 24-well plates (*n* = 10 per group) and exposed to varying concentrations of the compounds for 48 h. The control group was treated with 0.1% DMSO. The survival rates and morphological phenotypes were monitored at 24 h intervals (*n* = 10). (**D**) The LC_50_ of physcion, rhein and CPZ on the zebrafish larvae. (**E**) Representative images of neutrophil migration in the brain of the zebrafish larvae. (**F**,**G**) The numbers of neutrophil in the different groups were counted (*n* = 10). After injected LPS into the brain of the zebrafish larvae for 24 h, total RNA was extracted from the heads of 30 zebrafish larvae per group. Transcriptional analysis was conducted via RT-qPCR, where target gene levels ((**H**,**K**): *IL-1β*; (**I**,**L**): *IL-6*; (**J**,**M**): *TNF-α*) were normalized against the internal control *elfa* (*n* = 3). The quantitative results are expressed as mean ± SD; the statistical significance of the results was analyzed by one-way ANOVA. ### *p* < 0.001 versus the control group; *** *p* < 0.001, ** *p* < 0.01 and * *p* < 0.05 versus the LPS-treated group.

To evaluate the baseline safety of the selected compounds, toxicological screening was conducted on zebrafish larvae from 3 to 5 days post-fertilization (dpf). At the 5 dpf endpoint, larval mortality was strictly determined based on the complete absence of a visible heartbeat under a stereomicroscope. Physcion and CPZ exhibited minimal baseline toxicity, with their 48 h LC_50_ (lethal concentration 50) values determined to be greater than 80 µM (Figure 6A,C). In contrast, rhein exhibited pronounced developmental toxicity at higher concentrations, with a calculated 48 h LC_50_ of 25.04 µM (95% confidence interval [CI]: 23.4 to 27.28 µM) (Figure 6B). Notably, exposure to 40 and 80 µM of rhein induced severe phenotypic abnormalities prior to lethality; these abnormal phenotypes were predominantly characterized by gross morphological malformations, including spinal curvature, accompanied by localized hemorrhages in the head region. LPS could induce inflammatory responses at the site of injection in the brain of zebrafish larvae. Quantitative analysis in Figure 6E revealed a robust recruitment of neutrophils following LPS provocation. While CPZ (10 µM) exerted a clear suppressive effect, both physcion and rhein demonstrated a potent dose-dependent mitigation of this inflammatory cell infiltration, effectively restricting neutrophil mobilization to the site of injury (Figure 6F,G).

The transcriptional levels of pro-inflammatory mediators underlying the neuroinflammatory response in the LPS-injected zebrafish larvae were subsequently quantified utilizing qRT-PCR analysis. Significant induction of *IL-1β* and *IL-6* at the gene level was observed following LPS stimulation, while 10 µM CPZ obviously decreased these gene expressions compared with LPS-treated group. The LPS-mediated elevation of the *IL-1β*, *IL-6* and *TNF-α* transcripts was markedly attenuated by the administration of either physcion or rhein, demonstrating their potent anti-inflammatory efficacy (Figure 6H–M).

### 2.8. Physcion and Rhein Alleviated LPS-Induced Impairments in Locomotor Behavior in Zebrafish Larvae

**Figure 7:**
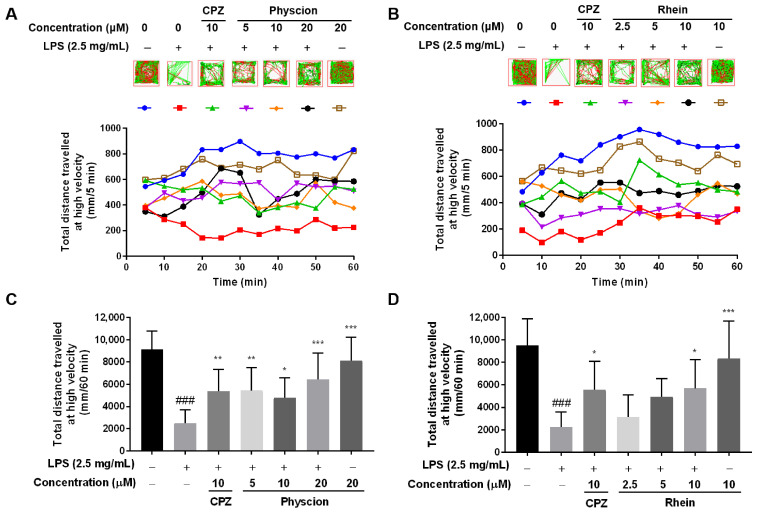
The effect of physcion and rhein on LPS-induced behavioral deficits in the zebrafish larvae. (**A**,**B**) The changes in total distance traveled at high velocity by period of locomotion behavior in LPS-induced zebrafish larvae pretreated with physcion (**A**) and rhein (**B**) (*n* ≥ 8). (**C**,**D**) Locomotor trajectory patterns in zebrafish larvae. Swimming tracks were captured in 5 min intervals with velocity-based chromatic visualization: black (<2 mm/s), green (2–20 mm/s), and red (>20 mm/s). The statistical quantification of total swimming distance at high velocity in LPS-challenged larvae following physcion (**C**) or rhein (**D**) intervention. The quantitative results are expressed as mean ± SD; the statistical significance of the results was analyzed by one-way ANOVA. ### *p* < 0.001 versus the control group; *** *p* < 0.001, ** *p* < 0.01 and * *p* < 0.05 versus the LPS-treated group.

To evaluate the protective potential of these compounds against LPS-triggered locomotor dysfunction in zebrafish larvae, we monitored the swimming behavior of zebrafish larvae through pretreatment with compounds and subsequent LPS injection. To rule out any intrinsic adverse effects on basal motor function, drug-only control groups were included. Zebrafish larvae treated with physcion or rhein exhibited no significant alterations in their baseline swimming behavior, confirming the safety of these compounds at the tested concentrations. As shown in Figure 7, the total distance traveled at high velocity of the LPS-treated group was clearly decreased compared with the control group. However, physcion at concentrations of 5, 10 and 20 µM significantly and dose-dependently reversed this dysfunction, markedly increasing the high-velocity travel distance (Figure 7A,C). Similarly, pretreatment with rhein at concentrations of 2.5–10 µM restored locomotor activity in a dose-dependent manner (Figure 7B,D). These behavioral data demonstrated that physcion and rhein possess significant in vivo neuroprotective efficacy against LPS-induced motor deficits, without intrinsically compromising normal physiological motility.

## 3. Discussion

The study aimed to discover novel candidates with potential functional TRPV1 inhibitory and anti-neuroinflammatory activities utilizing a structure-based virtual screening approach. This initial screening identified rhein and physcion as two promising candidates that significantly inhibit calcium influx mediated by TRPV1, suggesting their potential as functional inhibitors of TRPV1. Subsequent investigation into their anti-neuroinflammatory effects revealed that both compounds effectively mitigated neuroinflammation in LPS-stimulated BV2 microglial cells, which was closely paralleled by the downregulation of the Ca^2+^/CAMKK2/AKT and PI3K/AKT/NF-κB signaling pathways. Furthermore, in the LPS-injected zebrafish model, rhein and physcion both markedly reduced the mRNA expressions of key pro-inflammatory cytokines in the brain. Collectively, these findings indicated that physcion and rhein effectively mitigate microglial-mediated neuroinflammation associated with functional TRPV1 inhibition, thereby underscoring their clinical relevance as novel candidates for neurological disorder management.

In the present study, a structure-based virtual screening strategy was employed to identify potential TRPV1-modulating compounds from a library of over 200 small molecule compounds. To reduce false positive results, the compounds with the top 10% scores from the initial screening were selected for further clustering analysis. This process revealed that anthraquinones from rhubarb constituted the unique category, suggesting a shared structural basis underlies their potential interaction with TRPV1. To further assess the binding potential of anthraquinones, five representative anthraquinone compounds (rhein, physcion, aloe-emodin, emodin and chrysophanol) were analyzed using Uni-GBSA. Compared with docking scores alone, binding free energy estimation provides a more quantitative measure for ranking ligand–protein interactions. The Uni-GBSA analysis revealed differential binding affinities among the five anthraquinone compounds, with rhein and physcion exhibiting the most favorable binding free energies toward TRPV1.

Notably, the computational predictions were consistent with subsequent functional validation using calcium imaging assays. TRPV1 functions as a non-selective cation conductor, where its gating triggers intracellular Ca^2+^ influx. Given that TRPV1 activation facilitates substantial cation entry, fluorescence-based calcium imaging was utilized to monitor calcium transients in HEK293-hTRPV1 cells. Among the five tested anthraquinones, physcion and rhein exhibited the most significant inhibition of calcium influx mediated by TRPV1, indicating their potential as effective functional TRPV1 inhibitors. The findings suggested that physcion and rhein exert potent functional inhibitory activity against TRPV1, effectively suppressing channel-mediated calcium influx. This concordance between binding free energy estimation and functional results supports the predictive accuracy of virtual screening and molecular docking.

Previous research has demonstrated that emodin downregulated TRPV1 mRNA expression in sensory neurons, implying a potential interaction with this channel [32]. This may indicate that anthraquinones from rhubarb commonly exhibit the ability to modulate TRPV1, but direct functional evidence has been lacking until now. Physcion was shown to suppress LPS-induced TNF-α and IL-1β release and mitigate oxidative stress [33]. Separately, rhein was reported to inhibit neuroinflammation through interference with MAPK and IκB signaling [34]. While these studies established the phenotypic efficacy of the compounds, they did not probe the role of ion channel modulation in their mechanisms. Our study addressed these gaps by demonstrating that rhein and physcion function as potential functional inhibitors of TRPV1, as evidenced by calcium imaging and molecular docking. Furthermore, we delineated how the anti-neuroinflammatory responses elicited by these compounds are closely associated with the modulation of downstream calcium-dependent signaling pathways. Although emodin demonstrated comparable TRPV1 binding affinity in the initial molecular docking, it was ultimately excluded from subsequent in vivo testing primarily due to its weak functional inhibition of the CAP-evoked calcium influx. Furthermore, as observed in the cellular assays, the cytotoxicity of emodin may be intrinsically linked to its specific structural interactions or off-target mechanistic pathways. This potential mechanism-related toxicity represents a critical barrier for its therapeutic application. Incorporating this perspective highlights the structural and pharmacological advantages of physcion and rhein, which successfully achieve a safer balance between targeted functional TRPV1 modulation and cellular viability.

This study aligns with the current concept that TRPV1 is a context-dependent regulator of inflammation. Recent work demonstrated that LPS provocation enhances TRPV1 responsiveness, thereby amplifying calcium mobilization and reconfiguring pro-inflammatory cascades [35]. As a non-selective cation channel with high permeability to calcium, the activation of TRPV1 leads to calcium influx, thereby initiating a cascade of downstream signaling pathways. For instance, calcium influx facilitates AKT phosphorylation, which is crucial for AKT activation [36]. Phosphorylation of the IKK complex by AKT triggers NF-κB activation, promoting the production of pro-inflammatory signaling molecules [37]. As a result of IκBα phosphorylation, the NF-κB heterodimer composed of p65 and p50 moves into the nucleus, binding κB cis-acting sequences to drive the expression of pro-inflammatory mediators [38]. Therefore, in neuroinflammation, calcium acts as an upstream signal that can activate both CAMKK2 and the PI3K/AKT pathway. CAMKK2 contributes to AKT activation, while the PI3K/AKT signaling cascade induces NF-κB activation. The interplay between these pathways, influenced by calcium signaling and receptors like TRPV1, highlights the complexity of neuroinflammatory processes and potential therapeutic targets. In this study, the well-characterized TRPV1 antagonist CPZ was systematically employed as a positive reference control to evaluate and validate the anti-neuroinflammatory efficacy of physcion and rhein across both in vitro and in vivo models. Applying a similar LPS-induced neuroinflammatory model in microglia, we found that these functional TRPV1 inhibitors, rhein and physcion, potently suppressed this sensitized pathway. Our results demonstrated that physcion and rhein inhibit LPS-evoked Ca^2+^ mobilization, which was accompanied by the downregulation of downstream activation of the Ca^2+^/CAMKK2/AKT and PI3K/AKT/NF-κB signaling pathways, thereby attenuating the levels of NO and downregulating IL-6, IL-1β, and TNF-α secretion.

In the LPS-injected zebrafish larvae model, physcion and rhein significantly attenuated LPS-induced neutrophil migration to the brain and ameliorated associated behavioral impairments. This demonstrated their capacity to suppress neuroinflammation and its functional consequences at an organismal level. Our results reinforced the concept of TRPV1 as a context-dependent modulator of inflammatory signaling and further validated the therapeutic strategy of utilizing functional TRPV1 inhibitors like rhein and physcion to regulate Ca^2+^-dependent neuroinflammatory pathways.

Evaluating the therapeutic potential of physcion and rhein requires a careful assessment of their pharmacokinetics (PK) and toxicological profiles. For rhein, PK studies have shown that under compromised blood–brain barrier (BBB) conditions, the cerebrospinal fluid/plasma AUC ratio reaches ~17% [39]. For physcion, a comprehensive review noted its anti-inflammatory and neuroprotective activities, but also concluded that its PK profile remains incompletely characterized, and that dose-dependent hepatotoxicity has been reported [40]. Highly potent synthetic TRPV1 blockers have frequently failed in human trials due to severe on-target adverse events, particularly systemic hyperthermia and altered thermal sensation [41]. Importantly, the major clinical failure of AMG 517 was attributed to severe, dose-dependent hyperthermia in humans, arising from the critical physiological role of TRPV1 in body temperature regulation [42]. Naturally derived anthraquinones represent an alternative structural scaffold that may circumvent these severe thermoregulatory disruptions; notably, neither physcion nor rhein has been associated with hyperthermia in any published study. Nevertheless, dedicated thermoregulatory assessments and more complete pharmacokinetic and toxicity profiling, including measurements of unbound brain concentrations, are essential before advancing therapeutic claims.

Our study provides evidence supporting rhein and physcion as potential anti-neuroinflammatory agents associated with TRPV1 modulation; however, several limitations warrant explicit consideration. Primarily, although the molecular docking and calcium imaging assays strongly suggested functional TRPV1 inhibition, the precise biophysical mechanism of this interaction remains to be fully elucidated. The absence of direct receptor-binding assays or high-resolution electrophysiological measurements, such as whole-cell patch-clamp recordings, the possibility of indirect allosteric modulation, or off-target effects on parallel calcium pathways (such as TRPA1, IP3 receptors, or voltage-gated calcium channels), cannot be completely excluded. Secondly, future investigations must incorporate targeted genetic manipulations, such as siRNA-mediated knockdown or plasmid-driven overexpression of TRPV1 in BV-2 microglial cells, to strictly determine whether the anti-neuroinflammatory effects are exclusively dependent on this specific channel. Thirdly, although the preliminary in vivo zebrafish model provided basic safety data, rhein exhibited a narrow therapeutic window with its effective concentrations of 10 to 20 µM closely approaching the LC_50_ of 25 µM, whereas physcion displayed a much safer toxicity profile. Furthermore, although preliminary toxicological screenings confirmed normal morphology and survival at the maximum tested concentrations, the absence of drug-only controls in the neutrophil migration assay means potential basal effects on immune dynamics cannot be entirely excluded.

Despite these methodological constraints, the integration of computational screening with in vitro and in vivo functional validations provides a solid pharmacological basis for further exploring physcion and rhein. Moving forward, future research perspectives will focus on definitively validating the physical binding between compounds and the TRPV1 channel through advanced biophysical techniques. Furthermore, although the zebrafish model demonstrated the efficacy of both compounds in reducing neuroinflammation and behavioral deficits, a more direct mechanistic link to TRPV1 inhibition in vivo remains to be established in targeted genetic or mammalian models. Additional efforts will also be directed toward the structural optimization of these molecules to systematically expand their therapeutic safety windows. Ultimately, given that unresolved neuroinflammation serves as a primary driving force in the progression of a wide spectrum of CNS disorders, including various neurodegenerative diseases, discovering functional TRPV1 inhibitors from natural sources offers a highly valuable biochemical starting point. Further translational research building upon this framework may facilitate the development of targeted, safer therapeutic interventions for the clinical management of severe neurological conditions.

## 4. Materials and Methods

### 4.1. Reagents and Materials

CAP (Cat. No. HY-10448) and CPZ (Cat. No. HY-15640) were supplied by MCE (St. Louis, MO, USA). Physcion (Cat. No. WKQ-0000150), rhein (Cat. No. WKQ-0000151), emodin (Cat. No. WKQ-0000222) and aloe-emodin (Cat. No. WKQ-0000349) were obtained from Sichuan Weikeqi Biotech Co., Ltd. (Chengdu, China). Chrysophanol (Cat. No. CFN98751) was purchased from Wuhan Zhongbiao Tech Co., Ltd. (Wuhan, China). Stock solutions of CAP and CPZ were formulated in dimethyl sulfoxide (DMSO) at a concentration of 10 mM, and anthraquinones were similarly prepared at 100 mM. The solutions were kept at −20 °C, protected from light exposure.

LPS (Cat. No. L2880) and DMSO (Cat. No. D8418) were sourced from Sigma-Aldrich, based in St. Louis, MO, USA. Gibco (Thermo Fisher Scientific, Waltham, MA, USA) provided cell culture reagents, including Dulbecco’s Modified Eagle Medium (DMEM, Cat. No. 11965092), penicillin streptomycin (PS, Cat. No. 15140122), and phosphate-buffered saline (PBS, Cat. No. 10010023). Fetal bovine serum (FBS, Cat. No. 10091130), trypsin–EDTA (Cat. No. 25200-056), and TRIzol reagent (Cat. No. 15596026) came from Invitrogen (Carlsbad, CA, USA), whereas the Cell Counting Kit-8 (CCK-8, Cat. No. C0040) was obtained from Beyotime (Shanghai, China). SYBR^®^ Premix Ex Taq™ II kit (Cat. No. DRR820A) used for qPCR was supplied by TaKaRa (Dalian, China). All the other chemical reagents needed in the experiments were obtained from local sources.

### 4.2. Virtual Screening

Virtual screening was executed via the AutoDock Vina architecture (v. 1.2.0). To delineate the binding site within the TRPV1 receptor, the spatial coordinates of the co-crystallized antagonist capsazepine were utilized as a structural template, ensuring the docking grid encompassed the biologically relevant orthostatic pocket (PDB ID: 5IS0). A cubic grid box was constructed to enclose the entire active pocket, with dimensions set to 30 × 30 × 30 grid points (corresponding to approximately 24 Å × 24 Å × 24 Å, given the grid spacing of 0.8 Å in AutoDock Vina). To encapsulate the capsazepine-binding domain accurately, the search space was centered at the orthogonal coordinates x = 109.944, y = 93.765, and z = 104.958. During the virtual screening process, we optimized the search depth using an exhaustiveness setting of 32, a maximum energy range of 4 kcal/mol, and a limit of 10 binding modes per ligand. After completing the virtual screening, all small molecules were ranked in ascending order based on their binding affinity scores and their binding position.

### 4.3. Molecular Docking

Binding free energies between TRPV1 and the five selected anthraquinone compounds were further evaluated using the Uni-GBSA workflow. Uni-GBSA is an automated MM/GBSA-based platform that enables efficient estimation of ligand–protein binding free energies [43]. Following molecular docking, the TRPV1–ligand complexes were subjected to MM/GBSA calculations, and their binding free energies of five anthraquinones toward TRPV1 were calculated to allow a more refined assessment of their binding potential. The consensus docking poses were visualized using PyMOL v1.8, and the binding interactions were analyzed to compare the relative binding affinities of the ligands [44].

### 4.4. Cell Culture

The HEK293 and BV-2 cells (originally from ATCC) were propagated in complete DMEM (containing 10% FBS and 1% PS). The cultures were incubated under an atmosphere supplemented with 5% CO_2_ at a constant temperature of 37 °C, following established sterile technique protocols.

### 4.5. Transfection of TRPV1 Recombinant Plasmid in HEK293 Cells

The HEK293-hTRPV1 stable cells were established by delivering a human TRPV1 expression vector (NM_018727.5) into host cells via Lipo8000™-mediated lipofection. Briefly, the HEK293 cells were seeded in 6-well plates at a density of 5 × 10^5^ cells/well to reach 70–80% confluence prior to transfection. For each well, 2 µg of the plasmid DNA was complexed with the Lipo8000™ reagent (Beyotime Biotechnology, Shanghai, China) at a specific reagent-to-DNA ratio of 3:1 (*v*/*w*). The transfection complex was then incubated with the cells at 37 °C in a 5% CO2 humidified atmosphere, and the medium was carefully replaced with fresh complete medium 6 h post-transfection to minimize cytotoxicity. After an initial 48 h incubation, the growth medium was supplemented with puromycin to eliminate non-transfected populations. Stable integration was confirmed following limiting dilution cloning, which was employed to identify and propagate high-expressing cell clusters.

### 4.6. Calcium Imaging

Cytosolic calcium fluctuations were monitored using the Fluo-4 Direct™ Assay Kit (Invitrogen, USA). Briefly, the HEK293-hTRPV1 cells were seeded in 12-well plates and allowed to adhere for 24 h. Following a 4 h pre-incubation with anthraquinone derivatives (5, 10, and 20 μM) or CPZ (10 μM) as a positive control, the cells were loaded with 2 μM Fluo-4/AM for 30 min in a light-protected environment. The loading buffer consisted of a HEPES-buffered saline solution. After triple-washing to remove extracellular dye, real-time calcium transients were captured via an Olympus IX73 microscope equipped with the cellSens imaging system upon stimulation with 10 nM CAP. Data processing was conducted using the integrated cellSens v4.5 software suite.

### 4.7. Cell Viability Assay

To evaluate cytotoxicity, following overnight stabilization in 96-well plates, the initial culture medium was aspirated and replaced with low-serum DMEM (containing 0.5% FBS) containing various concentrations of physcion, rhein, or CPZ for a 24 h treatment period. Cellular metabolic activity, serving as a proxy for viability, was quantified using the CCK-8 reagent according to the manufacturer’s validated instructions.

### 4.8. Nitric Oxide Assay

The BV-2 microglial cells were cultured in 24-well plates until reaching confluence. To induce an inflammatory response, the cells were challenged with LPS (800 ng/mL) either alone or in combination with compounds. After 24 h, the accumulation of nitrite, a stable oxidative end-product of NO, was measured in the culture supernatants using a specialized Nitric Oxide Assay Kit (Beyotime, China) based on the Griess reaction principle.

### 4.9. Determination of Inflammatory Cytokines

To quantify the secretion of inflammatory mediators, the BV-2 microglia were stimulated with LPS (800 ng/mL) and varying concentrations of the test compounds (physcion or rhein), with CPZ included as a positive control, for 24 h. The extracellular release of IL-1β, IL-6 and TNF-α was then quantified using commercial ELISA kits (Invitrogen, USA). All the cytokine concentrations were normalized and expressed in pg/mL.

### 4.10. Western Blot Analysis

Total protein was extracted from the PBS-washed cells using supplemented RIPA lysis buffer (containing 1% PMSF and protease/phosphatase inhibitors) for 20 min on ice. Following SDS-PAGE separation and electrotransfer to PVDF membranes, non-specific epitopes were masked with 5% non-fat milk in TBST. After an overnight primary antibody challenge (4 °C), immunoreactive bands were developed by treating the blots with HRP-conjugated secondary antibodies (Beyotime, Cat. No. A0208, dilution 1:2000) for 1 h. Target protein bands were subsequently detected via ECL chemiluminescence and visualized for densitometric analysis. The primary antibodies, all supplied by Cell Signaling Technology, were utilized at specific dilutions as follows: anti-iNOS (Cat. No. 2982, dilution 1:1000), anti-CAMKK2 (Cat. No. 16810, dilution 1:1000), anti-p-CAMKK2 (Cat. No. 12818, dilution 1:1000), anti-PI3K (Cat. No. 4249, dilution 1:1000), anti-p-PI3K (Cat. No. 17366, dilution 1:1000), anti-AKT (Cat. No. 9272, dilution 1:1000), anti-p-AKT (Cat. No. 4060, dilution 1:1000), anti-IκBα (Cat. No. 4814, dilution 1:1000), anti-p-IκBα (Cat. No. 2859S, dilution 1:1000), anti-IKKα (Cat. No. 61294S, dilution 1:1000), anti-p-IKKα/β (Cat. No. 2697, dilution 1:1000), anti-NF-κB p65 (Cat. No. 4764, dilution 1:1000), anti-p-NF-κB p65 (Cat. No. 3033S, dilution 1:1000), and anti-β-actin (Cat. No. 4970S, dilution 1:1000).

### 4.11. Maintenance of Zebrafish Embryo and Larvae

This study utilized AB wild-type and transgenic Tg(*mpo*:EGFP) zebrafish strains, managed in adherence to the protocols outlined in the Zebrafish Handbook. All the animal procedures received formal clearance from the University of Macau Animal Research Ethics Committee (Permit No.: UMARE-021b-2020). Sexually mature fish were housed under a circadian photoperiod (14 h light/10 h dark) at 28.5 °C. Their nutritional regimen consisted of a daily serving of tropical fish flakes supplemented with two feedings of *Artemia salina* (brine shrimp) [45].

### 4.12. Toxicity Assay in Zebrafish Larvae

To assess the acute toxicity of the test compounds, healthy AB strain larvae at 3 days post-fertilization (3-dpf) were distributed into 24-well plates (*n* = 10 per well). The larvae were exposed to varying concentrations of physcion, rhein, or CPZ for a total duration of 48 h. The control groups were maintained in medium containing 0.1% DMSO. Viability was monitored at 24 h intervals to determine the survival trajectories and calculate the median lethal concentration (LC_50_) for each treatment group.

### 4.13. Zebrafish Larvae Morphological Observations

The Tg(*mpo*:EGFP) zebrafish model was utilized to monitor real-time neutrophil trafficking during inflammation [46]. Briefly, 3-dpf larvae (*n* = 12 per group) underwent a 48 h incubation with the specified compounds, using 0.1% DMSO and 10 μM CPZ as vehicle and positive controls, respectively. Following anesthesia with 0.02% tricaine, neuroinflammation was induced via intracerebral microinjection of LPS (2.5 mg/mL) using a Nanoject III system [47]. At 24 h post-injection, neutrophil migration and structural changes in the brain were visualized and captured via Leica fluorescence microscopy (Leika, Wetzlar, Germany).

### 4.14. Locomotion Behavioral Test

To evaluate behavioral changes, 3-dpf larvae were pretreated and challenged with LPS as previously described. Post-injection (24 h), individual larvae were transferred to 96-well plates for automated locomotor tracking (Viewpoint Life Science, Montreal, QC, Canada). Total swimming activity was monitored over a 60 min window, with the distance traveled recorded in 12 consecutive 5 min intervals to assess the recovery of neuromotor function across treatment groups.

### 4.15. RNA Isolation and qRT-PCR

For the larval model, 3-dpf zebrafish (*n* = 30 per group) underwent a 48 h compound exposure followed by intracerebral LPS stimulation (2.5 mg/mL). At 24 h post-injection, cephalic tissues were harvested for analysis. Simultaneously, BV-2 microglia were subjected to a 4 h co-incubation with LPS (800 ng/mL) and test ligands. Total RNA from both larval heads and cell lysates was isolated utilizing the TRIzol™ reagent (Invitrogen, USA). Following RNA extraction, first-strand cDNA synthesis was performed using a one-step reverse transcription system. Quantitative real-time PCR (qRT-PCR) was then executed on a QS7 System (Applied Biosystems, Carlsbad, CA, USA) employing SYBR^®^ Green chemistry. Transcriptional abundance was normalized to β-actin or elfα, with relative fold-changes determined via the (2^−ΔΔCt^) method [48]. Primer sequences are detailed in Table 2.

### 4.16. Data and Statistical Analysis

For each assay, at least three independent trials were conducted, with the quantitative findings expressed in the form of mean ± standard deviation (SD). Computational analysis was executed using GraphPad Prism 7.0 (La Jolla, CA, USA). To evaluate differences between multiple experimental cohorts, a one-way analysis of variance (ANOVA) followed by Dunnett’s post hoc test was implemented. A threshold of *p* < 0.05 was pre-specified to define statistical significance.

## 5. Conclusions

In conclusion, this study indicated that physcion and rhein could inhibit calcium influx mediated by TRPV1 and emerge as potential functional TRPV1 inhibitors. Meanwhile, in the BV2 microglial cells, physcion and rhein inhibited NO production, pro-inflammatory cytokine release triggered by LPS, which was accompanied by the downregulated mRNA expressions of *iNOS*, *IL-6*, *IL-1β* and *TNF-α*. Moreover, their anti-neuroinflammatory effects were closely associated with the suppression of the Ca^2+^/CAMKK2/AKT and PI3K/AKT/NF-κB signaling pathways. Furthermore, physcion and rhein showed inhibitory effects on LPS-induced neutrophil migration and ameliorated locomotor deficits, restoring the *IL-1β*, *IL-6* and *TNF-α* mRNA expressions in zebrafish larvae. These findings not only broaden the understanding of the pharmacological mechanisms by which anthraquinones derived from rhubarb mitigate neuroinflammation, but also highlight the therapeutic potential of targeting TRPV1-mediated calcium dysregulation in neurological disorders.

## Figures and Tables

**Table 1:** The binding free energy values of compounds with TRPV1.

Target	Compounds	Binding Energy (kcal/mol)
TRPV1	Rhein	−47.32
	Physcion	−46.37
	Chrysophanol	−44.50
	Aloe-emodin	−41.10
	Emodin	−39.40

**Table 2:** Primers used in qRT-PCR assay.

	Primer		Sequence
Mouse	iNOS	Forward	CCCTTCCGAAGTTTCTGGCAGCAGC
		Reverse	GGCTGTCAGAGAGCCTCGTGGCTTTGG
	IL-1β	Forward	TCCAGGATGAGGACATGAGCAC
		Reverse	GAACGTCACACACCAGCAGGTTA
	IL-6	Forward	CCGGAGAGGAGACTTCACAG
		Reverse	TCCACGATTTCCCAGAGAAC
	TNF-α	Forward	ACTGAACTTCGGGGTGATTG
		Reverse	GCTTGGTGGTTTGCTACGAC
	Β-actin	Forward	TTCGTTGCCGGTCCACACCC
		Reverse	GCTTTGCACATGCCGGAGCC
Zebrafish	IL-1β	Forward	CATTTGCAGGCCGTCACA
		Reverse	GGACATGCTGAAGCGCACTT
	IL-6	Forward	TCAACTTCTCCAGCGTGATG
		Reverse	TCTTTCCCTCTTTTCCTCCTG
	TNF-α	Forward	TAGAACAACCCAGCAAACTC
		Reverse	TCTCCTTCTTCAACATCCAA
	elfα	Forward	GCTCAAACATGGGCTGGTTC
		Reverse	AGGGCATCAAGAAGAGTAGTACCG

## Data Availability

Data are contained within the article and Supplementary Materials.

## Supplementary Materials

The following supporting information can be downloaded at https://www.mdpi.com/article/10.3390/molecules31122049/s1, Table S1: Virtual screening of small molecule compound library; Figure S1: Cytotoxicity assessment of five rhubarb-derived anthraquinones in HEK293-hTRPV1 cells. The cell viability was evaluated via the CCK-8 assay following a 4-h incubation with various concentrations of the compounds. (A–E) Concentration-dependent effects of (A) emodin, (B) aloe-emodin, (C) chrysophanol, (D) physcion, and (E) rhein on HEK293-hTRPV1 cell viability. (F) The calculated IC_50_ values for the five tested compounds. Data are expressed as mean ± SD (*n* = 3). Statistical significance of results was analyzed by one-way ANOVA. * *p* < 0.05, ** *p* < 0.01, and *** *p* < 0.001 versus the control group.

## Abbreviations

The following abbreviations are used in this manuscript:

BBB	blood–brain barrier
CAMKK2	calcium/calmodulin-dependent protein kinase kinase 2
CAP	capsaicin
CCK-8	Cell Counting Kit-8
CI	confidence interval
CNS	central nervous system
CPZ	capsazepine
TRPV1	the transient receptor potential cation channel subfamily V member 1
DMEM	Dulbecco’s Modified Eagle Medium
DMSO	dimethyl sulfoxide
dpf	days post-fertilization
FBS	fetal bovine serum
IC_50_	half maximal inhibitory concentration
IL-6	interleukin-6
IL-1β	interleukin-1β
iNOS	inducible nitric oxide synthase
LC_50_	lethal concentration 50
LPS	lipopolysaccharide
NF-κB	nuclear factor kappa-light-chain-enhancer of activated B cells
NO	nitric oxide
PBS	phosphate-buffered saline
PI3K	phosphoinositide 3-kinase
PK	pharmacokinetics
PS	penicillin streptomycin
ROS	reactive oxygen species
TNF-α	tumor necrosis factor-α
