# Supplementary material for: Discovery of Rhubarb Anthraquinones Physcion and Rhein as Functional Inhibitors of TRPV1 Against Lipopolysaccharide-Induced Neuroinflammation
Source: Molecules. 2026 Jun 11;31(12):2049. doi: 10.3390/molecules31122049 (PMC13304890; doi:10.3390/molecules31122049)
Supplement: Supplementary file 1 [file molecules-31-02049-s001.zip › molecules-4301721-supplementary.pdf]

Supplementary Table S1 Virtual screening of small molecule compound library.

Ligand_name	Scores
Rhein_110757-2002-6	10.378
Aloe-emodin_110795-200806	-9.825
Glimepiride_93479-97-1	-9.82
Gliquidone_33342-05-1	-9.815
Manidipine_hydrochloride_89226-75-5	-9.716
Progesterone_57-83-0	-9.638
Verbascoside_E-0226	-9.604
Depofemin_313-06-4	-9.598
Estradiol_benzoate_50-50-0	-9.581
N-[4-[2-(2-Amino-4,7-dihydro-4-oxo-1H-pyrrolo[2,3-d]pyrimidin-5-yl)ethyl]benzoyl]-L-glutamic_acid_disodium_salt_137281-23-3	-9.554
Phenolphthalein_77-09-8	-9.546
Physcion_110758-200610	-9.52
Clotrimazole_23593-75-1	-9.482
Emodin_110756-200110	-9.466
INDIRUBIN-3'-MONOXIME_10404	-9.466
Dihydrotanshinone_	-9.41
Folic_acid_59-30-3	-9.368
Sanguinarine_E-0278	-9.322
Ranolazine_95635-56-6	-9.277
Losartan_114798-26-4	-9.227
Indirubin_E-0228	-9.165
Phenol_Red_143-74-8	-9.163
triptolide_111567-200502	-9.061
Cepharanthine_481-49-2	-9.026
Polydatin_111575-200502	-8.957
berberine	-8.951
Erlotinib_hydrochloride_183319-69-9	-8.923
Cinobufagin	-8.92
Sulfinpyrazone_57-96-5	-8.874
Wilforlide_A_111597-200402	-8.873
CALYCOSIN_PRF15093002	-8.846
Formononetin_	-8.84
Chelerythrine_111718-200501	-8.806
(20α,22R,25S)-Spirosta-5-ene-3β-ol_	-8.759
chrysin_	-8.756
calycosin_05-2027	-8.738
Paeoniflorin_110736-200629	-8.72
Ultraviolet_Absorbent_UV-360_103597-45-1	-8.718
Cepharanthin_111647-200301	-8.687
Flubendazole_31430-15-6	-8.674

Schisandrin_C_		-8.661
Labetalol_hydrochloride_32780-64-6		-8.618
2-BENZOYLACETANILIDE_103-84-4		-8.568
Fangchinoline_110793-200504		-8.563
Piperine_0775-200203		-8.546
Nitidine_chloride_E-0111		-8.496
Sarsasapogenin_		-8.492
Linezolid_165800-03-3		-8.415
Ursolic_acid_110742-200516		-8.401
Honokiol_110730-200609		-8.383
Amodiaquin_dihydrochloride_dihydrate_6398-98-7		-8.381
Pefloxacin_149676-40-4		-8.37
PROFLAVINE_HEMISULFATE_1811-28-5		-8.354
Pioglitazone_hydrochloride_112529-15-4		-8.281
6-METHOXY-2-NAPHTHOIC_ACID_2471-70-7		-8.231
Oleanolic_acid_110709-200505		-8.221
alpha,alpha-Diphenyl-4-piperidinomethanol_115-46-8		-8.21
L-Tryptophan_73-22-3		-8.199
Tolperisone_hydrochloride_3644-61-9		-8.194
Carbenoxolone_471-53-4		-8.145
Cortisone_acetate_50-04-4		-8.132
Magnolol_110729-200510		-8.112
2-Hydroxy-N-(4-hydroxyphenyl)-benzamide_526-18-1		-8.064
Ginkgolide_C_		-8.032
FENDILINE_HYDROCHLORIDE_13636-18-5		-8.028
FENDILINE_HYDROCHLORIDE_13636_18_5		-8.017
maslinic_acid_		-8.009
Fluoxetine_54910-89-3		-8.002
Econazole_27220-47-9		-7.997
2-Amino-5-nitrobenzophenone_1775-95-7		-7.991
Homosalate_118-56-9		-7.991
Artemisinin_63968_64_9		-7.989
	1	-7.983
Ilexsaponin_A1_		-7.974
PHTHALYLSULFACETAMIDE_131-69-1		-7.964
Oridonin_111721-200501		-7.961
Miconazole_22916-47-8		-7.956
Rosiglitazone_122320-73-4		-7.954
Bupivacaine_hydrochloride_18010-40-7		-7.914
Betulin_Hi-18492		-7.907
Betulinic_acid_E-0349		-7.902
cycloastragenol_		-7.883
Sinensetin_E-0595		-7.873
OBB		-7.843

CLOMIPRAMINE_HCL_17321-77-6	-7.826
Rutundic_acid_	-7.808
Naphazoline_hydrochloride_550-99-2	-7.803
Indometacin_53-86-1	-7.791
Moxifloxacin_hydrochloride_186826-86-8	-7.79
3,5-Dinitrobenzamide_121-81-3	-7.771
Ginkgolide_A_	-7.767
Atazanavir_sulfate_229975_97_7	-7.73
Amiloride_hydrochloride_2016-88-8	-7.71
Octyl_4-methoxycinnamate_5466-77-3	-7.702
Dibenzothiophene_132-65-0	-7.691
Loganin_111640-200503	-7.69
Sinomenine_0774-200206	-7.672
Sodium_danshensu_110316	-7.656
trans-Resveratrol_70675	-7.654
Ginsenoside_Rg1_	-7.646
Resveratrol_	-7.638
3-HYDROXY-P-BUTYROPHENETIDINE_1083-57-4	-7.613
Homatropine_Hydrobromide_51-56-9	-7.601
Cinchocaine_85-79-0	-7.591
3,5,4'-Trimethoxystilbene_P8262K	-7.589
Benzethonium_chloride_121_54_0	-7.579
Atropine_sulfate_5908_99_6	-7.57
Menadione_58-27-5	-7.557
melatonin_211835	-7.552
Ginkgolide_B_	-7.531
Gomisin_A_	-7.515
Promethazine_hydrochloride_58-33-3	-7.496
Adiphenine_hydrochloride_50-42-0	-7.492
Astragaloside_IV_07-1008	-7.488
Protopanaxatriol_	-7.487
CYCLANDELATE_456-59-7	-7.457
Inosine_58-63-9	-7.411
GINSENOSIDE_F1_	-7.405
Tizanidine_64461-82-1	-7.391
Pentoxifylline_	-7.387
Dehydrocholic_acid_81-23-2	-7.386
Nitroxoline_4008-48-4	-7.382
Azelnidipine_123524-52-7	-7.377
Naringin_10236-47-2	-7.353
MEPHENESIN_59-47-2	-7.349
Ginsenoside_Rg6_	-7.313
ISORHYNCHOPHYLLINE_	-7.313
5,7-Dichloro-8-hydroxyquinoline_773-76-2	-7.284

Cefradine_38821-53-3	-7.274
Oxybenzone_131-57-7	-7.269
Atropine_51-55-8	-7.267
Atenolol_29122-68-7	-7.265
Doxofylline_69975-86-6	-7.24
Cilnidipine_132203-70-4	-7.217
Zonisamide_68291-97-4	-7.206
Halazone_80-13-7	-7.198
5-Methyl-2-phenyl-1,2-dihydropyrazol-3-one_89-25-8	-7.197
Ginsenoside_Rg3_	-7.188
Naringin_E-0032	-7.186
2-CHLORO-4-NITROBENZAMIDE_3011-89-0	-7.181
Ginsenoside-Rg5_	-7.172
Riluzole_1744-22-5	-7.144
Primidone_125-33-7	-7.13
Methylene_Blue_trihydrate_7220_79_3	-7.125
Octocrylene_6197-30-4	-7.121
Sedanolide_s-0653	-7.12
4-acetamidophenylacetic_acid_18699-02-0	-7.113
Nobiletin_E-0188	-7.109
Bilobalide_	-7.107
Ticlopidine_hydrochloride_53885-35-1	-7.084
Colchicine_64-86-8	-7.066
trans-ferulic_acid_1083087	-7.051
Tangeretin_	-7.033
Streptomycin_sulfate_3810_74_0	-7.024
2-Ethylhexyl_salicylate_118-60-5	-7.005
1-[3-(2,4,6-Trimethoxybenzoyl)propyl]pyrrolidinium_chloride_35543-24-9	-6.995
2-Methoxynaphthalene_2-Methoxynaphthalene	-6.995
D-(-)-Salicin_138-52-3	-6.976
Schisandrin_B_	-6.973
Acetylsalicylic_acid_50-78-2	-6.942
Gemcitabine_95058-81-4	-6.929
1-(2,6-Dichlorophenyl)-2-indolinone_15307-86-5	-6.905
Zileuton_111406-87-2	-6.878
20(S)-Ginsenoside_F2_	-6.874
Diphenhydramine_Hydrochloride_147-24-0	-6.862
Carzenide_138-41-0	-6.844
20(S)-Ginsenoside_Rh2_111748-200501	-6.837
Schisandrin_A_	-6.834
Notoginsenoside_R1_	-6.823
Dilthiazem_hydrochloride_33286-22-5	-6.756
8-Hydroxyquinoline_148-24-3	-6.733
Minoxidil_38304-91-5	-6.704

Acarbose_56180-94-0	-6.646
Procainamide_hydrochloride_614-39-1	-6.573
2-Ethoxybenzamide_938-73-8	-6.56
Sorafenib_tosylate_475207_59_1	-6.512
5,7-Diiodo-8-quinolinol_83-73-8	-6.507
Gallic_acid_11083-200302	-6.507
Benoxinate_Hydrochloride_5987-82-6	-6.499
Proparacaine_hydrochloride_	-6.492
Nimodipine_66085-59-4	-6.478
Acetaminophen_103-90-2	-6.443
3,5-Dihydroxybenzoic_acid_99-10-5	-6.429
3-Hydroxyphenyl_acetate_102-29-4	-6.388
4-Hexyl-1,3-benzenediol_136-77-6	-6.384
Methyldopa_555-30-6	-6.366
Crotamiton_483-63-6	-6.35
3,4-Dihydroxybenzoic_acid_99-50-3	-6.347
D-Sorbitol_50-70-4	-6.309
Synephrine_01-2008	-6.287
Methyl_salicylate_119-36-8	-6.262
2-(4-Chlorophenoxy)-2-methylpropionic_acid_882-09-7	-6.248
Amlodipine_Besylate_111470_99_6	-6.224
4'-Hydroxypropiophenone_70-70-2	-6.221
Tetrahydroxyquinone_319-89-1	-6.2
Dropropizine_17692-31-8	-6.176
Sodium_4_aminosalicylate_dihydrate_6018_19_5	-6.17
Nicotinic_acid_N-oxide_2398-81-4	-6.157
PROCAINE_59-46-1	-6.139
Schisandrin_	-6.126
Bromisoval_496-67-3	-6.102
Piracetam_7491-74-9	-6.052
Sulfanilamide_63-74-1	-6.01
Maltose_69-79-4	-6.001
Pargyline_555-57-7	-5.983
α-Lipoic_Acid_1077-28-7	-5.973
Dulcitol_608-66-2	-5.927
D-(+)-XYLOSE_25990-60-7	-5.885
Phenethyl_alcohol_60-12-8	-5.858
Adenine_73-24-5	-5.816
6-Mercaptopurine_50-44-2	-5.766
Alverine_citrate_5560-59-8	-5.759
Betahistine_mesylate_54856_23_4	-5.723
Betahistine_dihydrochloride_5579-84-0	-5.715
Ferrous_fumarate_141-01-5	-5.7
1,2,2,6,6-PENTAMETHYLPIPERIDINE_79-55-0	-5.659

DIHYDROTHYMINE_696-04-8	-5.594
TYLOXAPOL_25301-02-4	-5.568
Mannitol_69-65-8	-5.538
6-Acetamidohexanoic_acid_57-08-9	-5.512
Pheniramine_maleate_132_20_7	-5.46
Uracil_66-22-8	-5.414
Inositol_87-89-8	-5.382
LIGUSTRAZOINEHYDROCHLORIDE_	-5.369
1-Hydroxyethylidene-1,1-diphosphonic_acid_2809-21-4	-5.359
1,3-PROPANEDISULFONIC_ACID_DISODIUM_SALT_36589-58-9	-5.238
Citiolone_17896-21-8	-5.235
L-Isoleucine_73-32-5	-5.204
Bisoprolol_fumarate_104344_23_2	-5.164
3,5,5-TRIMETHYLOXAZOLIDINE-2,4-DIONE_127-48-0	-5.111
DL-GAMMA-AMINO-B-HYDROXYBUTYRIC_ACIDCRYS_TALLINE_924-49-2	-4.721
Stachydrine_hydrochloride_110712_200508	-4.708
Urethane_51-79-6	-4.388
Taurine_107-35-7	-4.185
2(5H)-Furanone_497-23-4	-4.178
Guanidine_hydrochloride_50_01_1	-4.111
Allylthiourea_109-57-9	-4.028
2-Aminothiazole_96-50-4	-3.897
Betaine_hydrochloride_590_46_5	-3.779
Piperazine_110-85-0	-3.737
Econazole_nitrate_24169-02-6	-3.636
Etamsylate_2624-44-4	-3.338
Etamsylate_2624_44_4	-3.335
Diclofenac_diethylamine_78213-16-8	-3.308
Dimethyl_sulfoxide_67-68-5	-2.74
Potassium_iodide_7681-11-0	-1.906
Ethylenediaminetetraacetic_acid_trisodium_salt_solution_150-38-9	-1.707
SULFAGUANIDINE_MONOHYDRATE_57_67_0	-1.68
D-(+)-Maltose_monohydrate_6363-53-7	-1.679
Cefadroxil_50370_12_2	-1.678
Quercetin_100081_200907	-1.678
Valethamate_bromide_90_22_2	-1.291
HEXAMETHONIUM_BROMIDE_55-97-0	-1.277

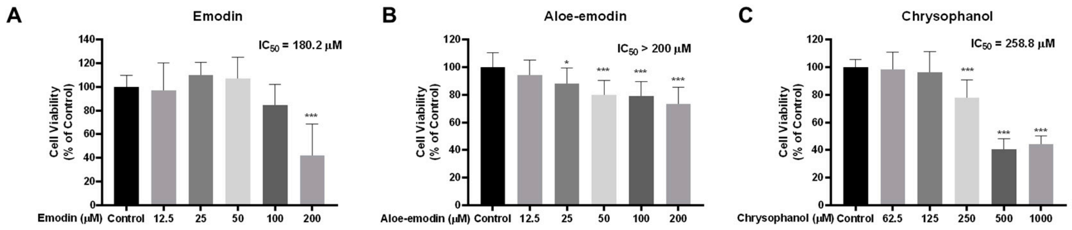

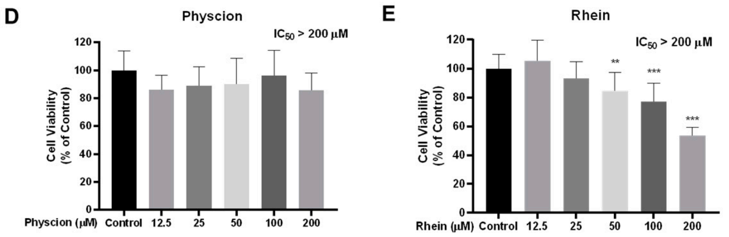

F

Anthraquinones	IC_{50} (μM)
Emodin	180.2
Aloe-emodin	> 200
Chrysophanol	258.8
Physcion	> 200
Rhein	> 200

Figure S1. Cytotoxicity assessment of five rhubarb-derived anthraquinones in HEK293-hTRPV1 cells. The cell viability was evaluated via the CCK-8 assay following a 4-hour incubation with various concentrations of the compounds. **(A–E)** Concentration-dependent effects of (A) emodin, (B) aloe-emodin, (C) chrysophanol, (D) physcion, and (E) rhein on HEK293-hTRPV1 cell viability. **(F)** The calculated half-maximal inhibitory concentration (IC_{50}) values for the five tested compounds. Data are expressed as mean ± SD ($n = 3$). Statistical significance of results was analyzed by one-way ANOVA. *$p < 0.05$, **$p < 0.01$, and ***$p < 0.001$ versus the control group.
